# Changing clinical characteristics of non-cystic fibrosis bronchiectasis in children

**DOI:** 10.1186/s12890-020-01214-7

**Published:** 2020-06-16

**Authors:** Ela Erdem Eralp, Yasemin Gokdemir, Emine Atag, Nilay Bas Ikizoglu, Pinar Ergenekon, Cansu Yilmaz Yegit, Arif Kut, Refika Ersu, Fazilet Karakoc, Bulent Karadag

**Affiliations:** 1 Division of Pediatric Pulmonology, Marmara University, School of Medicine, Istanbul, Turkey; 2 Division of Pediatric Pulmonology, Maltepe University, School of Medicine, Istanbul, Turkey

**Keywords:** Bronchiectasis, Child, Primary ciliary dyskinesia, Spirometry

## Abstract

**Background:**

The prevalence of non-cystic fibrosis (CF) bronchiectasis is increasing in both developed and developing countries in recent years. Although the main features remain similar, etiologies seem to change. Our aim was to evaluate the clinical and laboratory characteristics of our recent non-CF bronchiectasis patients and to compare these with our historical cohort in 2001.

**Methods:**

One hundred four children with non-CF bronchiectasis followed between 2002 and 2019 were enrolled. Age of diagnosis, underlying etiology and microorganisms in sputum culture were recorded. Clinical outcomes were evaluated in terms of lung function tests and annual pulmonary exacerbation rates at presentation and within the last 12 months.

**Results:**

Mean FEV1 and FVC %predicted at presentation improved compared to historical cohort (76.6 ± 17.1 vs. 63.3 ± 22.1 and 76.6 ± 15.1 vs. 67.3 ± 23.1, respectively; *p* <  0.001). There was a significant decrease in pulmonary exacerbation rate from 6.05 ± 2.88 at presentation to 3.23 ± 2.08 during follow-up (*p* <  0.0001). In 80.8% of patients, an underlying etiology was identified. There was an increase in primary ciliary dyskinesia (PCD) (32.7% vs. 6.3%; *p* = 0.001), decrease in idiopathic cases (19.2% vs. 37.8%; *p* = 0.03) with no change in postinfectious and immunodeficiencies as underlying etiology. Sputum cultures were positive in 77.9% of patients which was 46.9% in the historical cohort (*p* = 0.001).

**Conclusion:**

Baseline pulmonary function tests were better and distribution of underlying etiology had changed with a remarkable increase in diagnosis of PCD in the recent cohort.

## Background

Childhood bronchiectasis is a chronic pulmonary disorder defined as a clinical syndrome (persistent or recurrent [> 3] episodes of chronic wet or productive cough, sometimes with coarse crackles and digital clubbing), confirmed by the presence of bronchial dilation in high resolution chest tomography (HRCT) [1]. Although bronchiectasis has been called as an orphan disease in the past, it is now considered as one of the most common causes of chronic respiratory disease in both developed and developing countries [2].

The exact incidence of non-cystic fibrosis (CF) bronchiectasis in children is not known, but studies from England and New Zealand people of European heritage reported the incidence as 0.2/100,000 and 1.5/100,000 per year respectively; whereas incidence was highest in Pacific children as 17.8/100.000 [3, 4]. Main etiologies for non-CF bronchiectasis are infections, immune deficiencies, primary ciliary dyskinesia (PCD), and aspirations, but in 34% of patients no underlying cause was identified [5]. Both in developed and developing countries, postinfection was the leading etiology of non-CF bronchiectasis in the past years [3]. Highest incidences were reported in socially disadvantaged populations of developed countries as 90% by Chang et al. from Australia and 93% by Singleton et al. from Alaska [6, 7]. Brower et al. in a review including 12 studies found recurrent lower respiratory tract infections as the most common underlying cause (61%), followed by measles (14%), tuberculosis (11%) and pertussis (5%) [5]. With improvement in vaccination programs, prevention of diseases like measles and pertussis, effective tuberculosis control programs, improved access to health care and effective treatment of lower respiratory tract infections incidence of postinfectious bronchiectasis decreased to 7-12% in developed countries [8–10]. Immune deficiencies (10-34%) and PCD (2-24%) as the underlying reason were generally reported more frequently in developed countries with no change in the frequency over the years [8–13]. Idiopathic cases did not seem to change in frequency in both developed and developing countries in the last years. Kapur et al. in a recent study found no underlying etiology in 55% of patients in a developed country [9]. Also in a study conducted with 80 children from India by Kumar et al., 36% of cases were classified as idiopathic [14].

Although there is a decrease in post infectious bronchiectasis in developed countries, overall prevalence of non-CF bronchiectasis appears to be increasing particularly in adults [15, 16]. This increase in prevalence may be related to improved diagnostic rates secondary to improved awareness and improved access to HRCT scans.

We previously reported clinical features of non-CF bronchiectasis followed in our division between 1987 and 2001 [17]. To our knowledge, changes in general characteristics of patients with non-CF bronchiectasis in time in the same center have not been reported previously. We hypothesized that the underlying etiology and clinical characteristics of non-CF bronchiectasis changed over time. Our aim was to evaluate the changing characteristics of non-CF bronchiectasis in a decade comparing the recent cohort of patients with historical controls.

## Methods

This was an observational study including two cohorts from the same center. Recent cohort consisted of non-CF bronchiectasis patients (< 18 years of age) who have been followed in the Marmara University Pediatric Pulmonology clinic between 2002 and 2019. Historical cohort consisted of patients enrolled in our previous study which was done between 1987 and 2001. There were no overlapping patients between two cohorts. Study was approved by the local ethics committee and written informed consent was taken from the families.

**Fig. 1:**
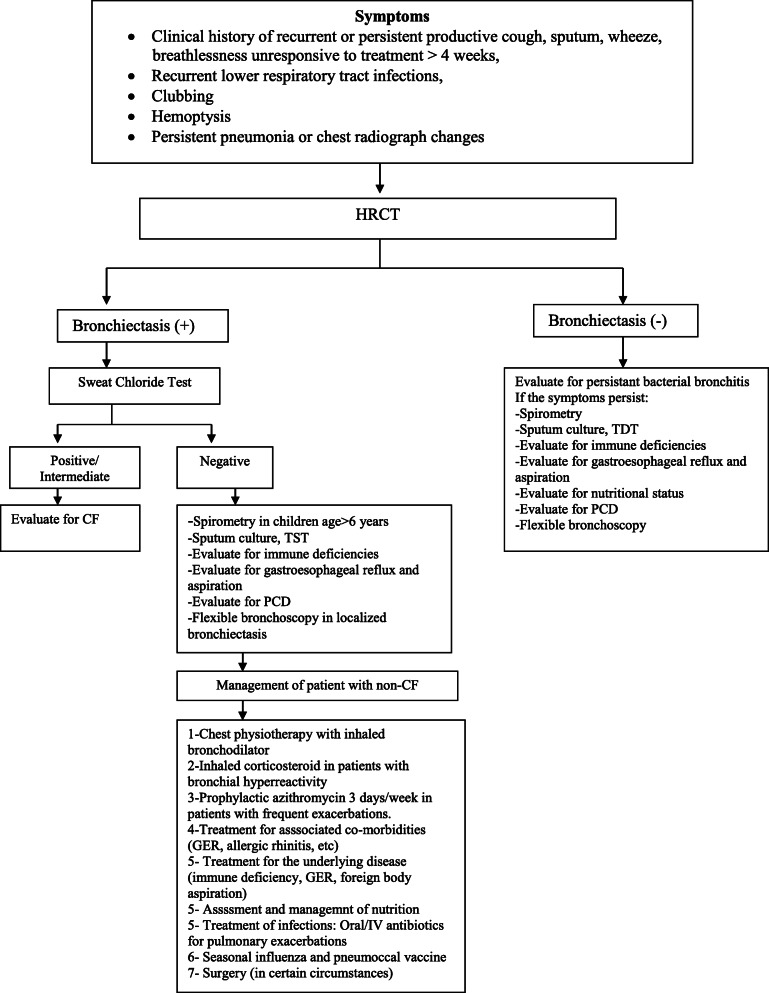
Algorithm for diagnosis and treatment of non-CF bronchiectasis

Patients with a clinical history of recurrent or persistent productive cough, sputum, wheeze, breathlessness unresponsive to treatment and recurrent lower respiratory tract infections were evaluated for bronchiectasis (Fig. 1) [18]. Diagnosis of bronchiectasis confirmed by the HRCT obtained during an asymptomatic period and at least 3 months after the last infectious exacerbation. Bronchiectasis was diagnosed if there was evidence of bronchial dilation (internal bronchial diameter greater than the accompanying pulmonary artery) and a lack of bronchial tapering on sequential slices [19]. The bronchi were evaluated on a lobar basis (regarding the lingula as a separate lobe). Bronchiectasis was defined as localized if only one lobe, and multilobar if more than one lobe was was affected.

### Study design

Patients were followed up at 3 months intervals. At each visit, patients or parents were asked about the presence of symptoms (cough, sputum, wheezing, dyspnea, hemotysis) and frequency of antibiotic use since last visit. Demographic, clinical and laboratory data including gender, age at the of onset of symptoms, duration of symptoms before diagnosis, age at diagnosis, presence of consanguinity, presenting symptoms; history of previous lower respiratory infections including pertussis, measles, varicella, tuberculosis before the diagnosis; number of lower respiratory tract infections within the previous 12 months at presentation and within the last 12 months of the follow-up requiring oral or intavenous antibiotics or hospitalization, history of surgery for bronchiectasis, localization of bronchiectasis on chest CT scan and pulmonary fuction test results were retrieved from medical records. Pulmonary exacerbation was defined by the any of the following; change in cough quality from dry to wet and/or sputum production for ≥3 days, breathlessness, chest pain, crepitations, wheeze with or without an increase in values of infectious markers [20, 21]. The presence of clubbing, chest deformities and nasal polyps were noted on physical examination. For microbiological evaluation, sputum samples were obtained when possible and cultured for bacteria.

For the etiological work-up of bronchiectasis, immunological evaluation including IgG, M, A and E, IgG subclass levels, specific antibody responses to tetanus toxoid, capsular polysaccharides of *Streptococcus pneumonia* and surface antigen of Hepatitis B virus, T lymphocyte subsets and neutrophil function tests were measured in all patients. In order to exclude CF, sweat test and genetic analysis (if required) were performed and patients with positive sweat tests (chloride levels > 60 mEq/l) or two CF mutations were excluded from the study.

Postinfectious etiology was evaluated by asking history of lower respiratory infections including measles, pertussis, varicella and tuberculosis. We performed tuberculin skin test (TST) to on all patients and patients with positive TST were re-evaluated further for tuberculosis.

PCD was diagnosed by the electronic microscopic evaluation of nasal cilia biopsy and/or positive immunofluorescence staining and/or decreased nasal nitric oxide level measurement and/or the presence of dextrocardia with the typical findings of PCD and PICADAR score > 5. Nasal nitric oxide cutoff value for PCD was defined at 77 nl/minute [22]. In patients with a decreased nasal nitric oxide levels but a negative electronic microscopic result or in whom evaulation could not be done, immunofluorescence staining was performed in Muenster University Hospital. Antibodies against DNAH5, GAS8, DNAH11 and RSPH9; in some selected cases DNALI1 and CCDC39 were asessed.

Asthma was diagnosed if patients had the history of wheeze, shortness of breath, chest tightness and/or cough and by variable expiratory airflow limitation according to Global Initiative for Asthma (GINA) [23]. In order to differentiate asthma from bronchiectasis patients with bronchial hyperreactivity, patients followed at least for 2 years before the diagnosis of bronchiectasis in a pediatric allergy or pulmonology clinic without any abnormality in chest x-ray or HRCT before, were labelled as asthmatic. Patients without an underlying etiology for bronchiectasis were classified as idiopathic bronchiectasis.

Spirometry (MIR Srl Spirobank, Rome, Italy) was performed according to the criteria of the American Thoracic Society by all children who were able to cooperate. Measurements included forced vital capacity (FVC) and forced expiratory volume in 1 s (FEV1); values were expressed as percentage of the predicted normal values [24]. Measurements were repeated 15 min after inhalation of 200 microgram salbutamol metered dose inhaler via a spacer (Volumatic, Allen & Hanburys, Uxbridge, UK). A positive bronchodilator response was defined as an FEV1 improvement of more than 12% of baseline [25].

### Statistical analysis

Statistical analysis was carried out with with SPSS for Windows version 20.0. Continuous variables were described through means, standard deviations and medians, whereas categorical variables were presented as frequency and percentages. Categorical variables were compared with Chi-square or with Fisher’s exact test when 20% of the expected frequencies were less than five. Continuous variables among two groups were compared with Mann–Whitney U test, since the data did not follow a normal distribution. Continous variables for paired groups were compared through Wilcoxon test. Results were evaluated in 95% confidence interval and significance level was set at *p* value of 0.05.

## Results

### Demographic characteristisc

Recent cohort included 104 patients diagnosed as bronchiectasis after 2002 (45% male) with a median age of 8 years (range, 0. 1-16.5) at presentation.

In the recent cohort, median age of diagnosis was 7 years. The most common presenting symptom was cough (95.2%) followed by sputum (77.9%), wheezing (42.3%) and dyspnea (51%). Table 1 shows the general characteristics of two cohorts. Rate of clubbing was decreased, baseline FEV_1_ and FVC values were higher in the recent cohort when compared to the historical one (Table 1).

**Table 1:** Demographic data for non-CF patients

	Historical Cohort 1987–2001 (n:111)	Recent Cohort 2002–2019 (n:104)	*p*-value
Male gender n(%)	56 (50.5)	47 (45)	0.52
Age of onset of symptoms (yr), median (min-max)	1.5 (0–11.9)	1 (0–16)	0.11
Duration of symptoms before diagnosis (yr),			
median (min-max)	4 (0.1–14.9)	4 (0–15.5)	0.89
Age at presentation (yr), median (min-max)	7 (1–17.5)	8 (0.1–16.5)	0.36
Hemoptysis n(%)	11 (10)	7 (6.7)	0.40
Clubbing n(%)	45 (40.9)	22 (21.2)	**0.02**
Chest deformity,n(%)	16 (14.5)	12 (11.5)	0.53
Annual lower respiratory tract infection rate (mean ± SD)	6.6 ± 4.0	6.1 ± 2.9	0.91
Spirometry n:93 (mean ± SD)			
FEV1 (% predicted)	63.3 ± 22.1	76.6 ± 17.1	< **0.001**
FVC (% predicted)	67.3 ± 23.1	76.7 ± 15.1	< **0.001**

In terms of pulmonary function, there was an increase in the mean FEV_1_ and FVC (*p* <  0.001) in the recent cohort. Similar to the historical cohort, annual lower respiratory tract infection rate decreased from 6.1 ± 2.9 at presentation to 3.2 ± 2.1 during follow-up (*p* <  0.0001). There was no change between the baseline and last FEV_1_% in follow-up in the recent cohort (76.6 ± 17.1 vs. 76.96 ± 18.1; *p* = 0.91) which increased in the historical one (63.3 ± 22.1 vs. 75.2 ± 25.2; *p* <  0.001).

### Etiology

Underlying etiology was identified in 80.8% (n:84) of the patients in the resent cohort (Table 2). Most common cause of non-CF bronchiectasis was PCD (32.7%), followed by post-infectious (26%) and immunodeficiencies (17.3%). All of the PCD patients (n:34) had typical findings of PCD and PICADAR score > 5. In 26 of patients nasal nasal nitric oxide measurement, in 4 transmission electron microscopy, in 19 immunofluorescence staining (7 with outer dynein arm defect, 4 with inner dynein arm defect, 4 with nexin-dynein regulatory complex defect and 2 with radial spoke defects) and in 4 genetic analysis (2 patients had CCDC40 homozygous, 1 CCNO homozygous and RSPH4A homozygous mutations) were performed [26]. There was a significant increase in the frequency of PCD (6.3% vs. 32.7%, *p* = 0.001), and decrease in idiopathic cases (37.8% vs. 19.2%, *p* = 0.03) compared with the historical cohort. In the recent cohort, there was no patient with a history of foreign body aspiration. One patient with esophageal atresia and tracheoesophageal fistula and two patients operated for complex cardiac disease were classified in other group.

**Table 2:** Underlying etiologies for non-CF patients

	Historical Cohort 1987–2001	Recent Cohort 2002–2019	*p* value
Idiopathic	42 (37.8)	20 (19.2)	0.03
Postinfectious	33 (29.7)	27 (26)	0.43
Immunodeficiencies	17 (15.3)	18 (17.3)	0.69
PCD	7 (6.3)	34 (32.7)	0.001
Asthma	5 (4.5)	3 (2.9)	0.72
Foreign body aspirations	4 (3.6)	0	NA
Others	3 (2.7)	3 (2.9)	0.64
Esophageal atresia-tracheoesophageal fistula	3 (2.7)	1 (0.9)	
Cardiac diseases	0	2 (2)	

Values in parentheses are percentages

*NA* not applicable

### Localization of bronchiectasis

Localization of bronchiectasis was similar to the historical cohort, and left lower lobe was the most affected lobe in both groups. In 38.5% of the patients there was one lobe involvement, mostly the left lower lobe (21.2%) similar to the historical cohort (*p* = 0.75). Although statistically insignificant, multilobar involvement tended to decrease (31.9% vs. 21.4%, *p* = 0.26) and bilobar involvement trended to increase (28.1% vs 41.3% (*p* = 0.09) in the recent cohort compared with the historical one.

### Sputum culture

Rate of positive sputum culture increased in the recent cohort (46.9% vs. 77.9%, *p* = 0.001). Most frequently isolated organisms were *H.influenza* (71.8%), *S.pneumonia* (47.1%), *M.catarrhalis* (14.4%), *P.aeruginosa* (11.5%) and *S.aureus* (11.5%), with the first three being significantly more common compared to the historical cohort (*p* = 0.001, *p* = 0.001 and *p* = 0.03, respectively) (Table 3).

**Table 3:** Distribution of microorganisms in sputum culture

	Historical Cohort 1987–2001	Recent Cohort 2002–2019	*p* value
None	53.1%	22.1%	< 0.001
*Hemophilus influenza*	38.5%	71.8%	< 0.001
*Streptococcus pneumonia*	23.0%	47.1%	< 0.001
*Staphylococcus aureus*	16.9%	11.5%	0.70
*Pseudomonas aeruginosa*	10.8%	11.5%	0.17
*Moraxella catarrhalis*	2.7%	14.4%	0.002
*Acinetobacter spp.*	None	3.8%	NA
*Stenotrophomonas maltophilia*	None	3.8%	NA
*Klebsiella pneumonia*	4.6%	1.9%	0.44

Values are percentages

*NA* not applicable

With regard to the treatment approach, surgery rate was decreased in the recent cohort (23.4% vs. 6.7%, *p* = 0.001).

## Discussion

This study describes the changes in the patient characteristics and etiology of bronchiectasis in chidren from a single tertiary reference center beween 2002 and 2019. There were significant differences between the pulmonary function tests at diagnosis and follow-up, underlying etiology, and rate of positive sputum microbiology between the recent and historical cohorts.

Diagnosis of bronchiectasis is often delayed worldwide [2, 3]. In a study from Italy, Santamaria et al. reported that the median age at diagnosis was 7 years though the children were symptomatic from the median age of 6 months [10]. In developed countries late referral to specialist or misdiagnoses are the most common reasons for diagnostic delay. In the our recent cohort median age at diagnosis was 7 years which was same with our historical cohort. Three different studies from Turkey and studies from indigenous populations also reported similar age at diagnosis (6. 2-8.5 years) [27–30]. In developing countries lack of resources and difficulty in accessing medical services are the most probable causes for the delay in diagnosis [31].

Spirometry results of bronchiectatic patients differ between the developed and developing countries. Lung functions of children from the developed countries were normal or near normal on diagnosis and stayed stable longitudinally [10, 32, 33]. In a study conducted among 991 PCD patients from International PCD cohort, mean FEV_1_ and mean FVC were lower than the mean reference values in all age groups with best lung function in children aged 6-9 years and the worst in adults [34]. Patients diagnosed at an early age had better lung function and milder disease [32]. A follow-up study enrolling Alaskan Native children showed that patients with bronchiectasis had significantly lower FEV_1_/FVC ratios than the chronic suppurative pulmonary patients without bronchiectasis [35]. In our recent cohort, baseline FEV_1_ values were higher compared to the historical cohort. Although, there was an increase in FEV_1_ during the follow-up in the historical cohort, there was no change in the recent cohort. Early and intensive treatment of bronchiectasis has been shown to prevent decline in FEV_1_ [1, 18, 32]. Kapur et al. also reported that pulmonary functions remained stable in non-CF bronchiectasis patients with a mean FEV_1_ of 76.8 ± 20.1% of predicted after 5 years follow up [32].

Rate of clubbing was significantly lower in the recent cohort compared to the historical cohort supporting the presence of a milder form of bronchiectasis. Rate of clubbing in non-CF bronchiectasis varies with a ratio of 20. 7-52% and it is more common in developing countries [29, 36, 37]. In a study conducted in non-CF bronchiectatic patients, 52% had digital clubbing and patients with digital clubbing had more extensive bronchiectasis; no association with pulmonary function tests were seen [37]. In the recent cohort, there was no association with the clubbing of the fingers and lung functions or severity of the bronchiectasis. Although the age of diagnosis did not differ, presence of better baseline pulmonary functions, decreased incidence of clubbing and decrease in follow-up exacerbation rates compared to baseline rates may suggest patients in the recent cohort had a milder form of bronchiectasis. Possible explanations for these changes may be due to increased annual income, better vaccine coverage, earlier recognition and referral and improved access to health care.

In children with bronchiectasis, an underlying disease process is identified in 63% of cases as shown in a systematic review of 12 studies including 989 children [5]. Previous pneumonia (17%), primary immunodeficiency (16%), recurrent aspiration, including inhaled foreign body (10%), and PCD (9%) are among the most common underlying etiologies. In developed contries, immunodeficiency is more commonly observed as the underlying disease in 9-34% of patient with bronchiectasis [4, 8–13, 30, 37, 38]. In developing countries bronchiectasis consequent to previous infection is more common, is the cause in 17 to 28% of cases [27–29, 39]. An important difference between developed and developing countries in terms of etiology is PCD which is higher in developed countries (15-23.8%) [10, 12]. In our recent cohort, 32.7% of patients with non-CF bronchiectasis were diagnosed as PCD which was significantly higher compared to the historical cohort. Bahceci et al. reviewed 110 non-CF BE patients between 200 5-2015 and compared them with their previous data for underlying etiology. They reported that the frequency of asthma and tuberculosis decreased but PCD (26.4%) and primary immune deficiency had increased in 10 years [28]. Underlying etiologies of non-CF bronchiectasis can be detected due to increased availability of diagnostic testing. In our cohort only in 19.2% of the patients underlying etiology could not be identified which is similar with the studies from developed countries [12, 13]. Recent developments in diagnostic testing such as nasal nitric oxide, electron microscopy, high speed videomicroscopy, immunoflorescence and genetic analysis enabled an accurate diagnosis of PCD earlier.

Bronchiectasis has been reported to have multilobar involvement in most pediatric studies [10, 12, 27, 29, 36]. Kapur et al. reported 73% children as having bilateral disease in their cohort of 52 children [32]. Multilobar disease predominated with a rate of 71% in a study from Saudi Arabia [36]. In our study group, multilobar involvement was lower. Although, there was a trend in involvement of lobes (less multilobar, more bilobar) between the historical and the recent cohorts, it did not reach statistical significance.

The British Thoracic Society bronchiectasis guideline emphasizes microbiological assessment for evaluating airway colonisation and infection [40]. Although there is limited data from developing countries, distribution of micro-organisms seems to be similar worldwide [1, 17, 31, 36, 39]. Studies from developed countries showed that *H. influenzae*, *S. pneumoniae* and *M. catarrhalis* are the major pathogens, whereas patients were colonized with *Pseudomonas aeruginosa* in 5–16% of children [41]. In our recent cohort, 22.1% patients had negative sputum cultures, which was significantly lower compared to the historical cohort. Identification of *H. influenzae*, *S. pneumoniae* and *M. catarrhalis* in sputum cultures were increased in the recent cohort. Number of positive sputum cultures may be due to the better qualified staff and equipment in microbiology laboratories leading to more accurate laboratory identification of the microbiology results.

This study had two limitations; it was conducted in a single reference center and as a tertiary referral centre, many patients with suspicion of PCD were referred to our center which might have caused the higher proportion of PCD patients.

## Conclusion

This study highlights the changing underlying aetiology and/or better diagnostic testing of pediatric non-CF bronchiectasis in a developing country setting. We have demonstrated a better lung function results, higher incidence of PCD, decreased incidence of idiopathic cases. An early diagnosis of underlying etiology is essential not only to improve the course and prognosis of disease, but also to prevent a progressive decline in lung function. As the availability of non-invasive and effective diagnostic technologies increase, the detection rate of underlying etiology will increase and improve the outcomes of non-CF bronchiectasis.

## Data Availability

The datasets used and/or analysed during the current study are available from the corresponding author on reasonable request.

## Abbreviations

CF: Cystic fibrosis
PCD: Primary ciliary dyskinesia
HRCT: High resolution chest tomography
TST: Tuberculin skin test
FVC: Forced vital capasity
FEV1: Forced expiratory volume in 1 s
